# Primary angle-closure glaucoma with goniodysgenesis in a Beagle dog

**DOI:** 10.1186/s12917-019-1812-1

**Published:** 2019-03-04

**Authors:** Shin Ae Park, Dodd Sledge, Colleen Monahan, Joshua T. Bartoe, András M. Komáromy

**Affiliations:** 1 Department of Small Animal Clinical Sciences, Veterinary Medical Center, College of Veterinary Medicine, Michigan State University, 736 Wilson Road, East Lansing, MI 48824 USA; 2 Michigan State University Veterinary Diagnostic Laboratory, Lansing, MI USA; 3 MPI Research, Mattawan, MI USA

**Keywords:** Canine, Beagle, Goniodysgenesis, Pectinate ligament dysplasia, Primary angle-closure glaucoma (PACG)

## Abstract

**Background:**

Open angle glaucoma is the only type of primary glaucoma reported in Beagles. This case report describes a primary angle-closure glaucoma in a Beagle and its diagnostic and prognostic relevance.

**Case presentation:**

A 12-year-old, neutered male Beagle presented to the Michigan State University (MSU) Comparative Ophthalmology Service for evaluation of suspected visual impairment. Complete ophthalmic examination of the left eye (OS) revealed: blepharospasm, absent menace response, moderate episcleral congestion, mild diffuse corneal edema, mydriasis, asteroid hyalosis, decreased myelination and cupping of the optic nerve head, and mild retinal vascular attenuation. Examinations of the right eye (OD) were within normal limits. Intraocular Pressure (IOP) were 24 mmHg OD and 49 mmHg OS. Gonioscopy OD revealed a narrow iridocorneal angle with moderate pectinate ligament dysplasia characterized by broad-based pectinate ligament strands (fibrae latae) and solid sheets (laminae) throughout all 4 quadrants. DNA testing revealed that the dog did not carry the Gly661Arg *ADAMTS10* mutation responsible for primary open angle glaucoma (POAG) in Beagles. The OS was medically managed with latanoprost 0.005% and dorzolamide HCl 2% /timolol malate 0.5% ophthalmic solutions for 7 months and then enucleated due to uncontrolled IOP. Histopathologic evaluation was consistent with goniodysgenesis with a broad, non-perforate, sheet-like band of uveal stroma bridging from the base of the iris to the terminal arborization of Descemet’s membrane. Approximately 14 months from the initial diagnosis of glaucoma OS, OD also developed glaucoma and was enucleated. Histopathologic findings were consistent with goniodysgenesis OD.

**Conclusions:**

To our knowledge, this is the first reported case of PACG with goniodysgenesis in a Beagle supported by clinical, genetic, and histopathologic data. It highlights the importance of gonioscopy in Beagles with glaucoma. Further studies with a larger number of dogs are warranted to characterize clinical manifestations and inheritance of PACG in this breed.

## Background

Glaucoma refers to a group of multifactorial diseases characterized by progressive axonal degeneration and death of retinal ganglion cells [1, 2]. A major risk factor in all veterinary species is elevated intraocular pressure [1]. Based on possible etiology, canine glaucoma can be classified as congenital, primary, or secondary [2]. Congenital glaucoma develops within the first few month of life [1]. Primary glaucoma is caused by inherent defects of the aqueous drainage pathway without clinically detectable concurrent ocular disease [1]. In secondary glaucoma, IOP elevation is associated with concurrent ocular disease, such as uveitis, neoplasia, lens luxation, and trauma [2]. Primary glaucoma is suspected to be inherited in over 45 canine breeds [3–6], and, based on the appearance of the iridocorneal angle (ICA), is subdivided into open and narrow/closed angle glaucoma [1].

Primary glaucoma in Beagles was first reported by *Gelatt* et al. in 1971, and has been extensively studied thereafter [7–13]. Glaucoma affects approximately 1% of Beagles in the U.S. [3]. Affected dogs initially have open ICAs in the early disease stages until the ciliary cleft collapses with end-stage glaucoma [14]. Hence, primary glaucoma in Beagles is classified as primary open-angle glaucoma (POAG). [9, 14] POAG in the Beagle is inherited as an autosomal-recessive trait [12] and has been reported to be caused by a Gly661Arg missense mutation of the metalloproteinase *ADAMTS10* gene [6, 15]. While there have been numerous publications on the clinical manifestations, structural changes, inheritance, genetics, and pathophysiology of POAG in the Beagle over the past four decades [8–13], to the best of our knowledge, there has been no report on primary angle-closure glaucoma (PACG) in this breed. The present case report describes a Beagle with PACG and goniodysgenesis diagnosed by clinical evaluations and histopathology.

## Case presentation

A 12-year-old, neutered male Beagle was referred to the Comparative Ophthalmology Service at MSU-VMC for evaluation of suspected visual impairment. The patient had trained and competed dog agility which allowed the owner to detect vision deficits early. Three weeks prior to the visit to MSU-VMC, the owner first noticed that the dog became slow to read hand signs on his left side. He was reported to be healthy otherwise and was not on any medication prior to the first visit to MSU. At the time of visit, a complete ophthalmic examination was performed including neuro-ophthalmic evaluation, Schirmer tear test (Schirmer tear test strips, Schering-Plough Animal health, Kenilworth, NJ, USA), fluorescein staining (Ful-Glo fluorescein sodium ophthalmic strips, AkornLake Forest, IL, USA), tonometry (Icare Tonovet, Vantaa, Finland), slit-lamp biomicroscopy (Kowa SL-17 portable slit lamp, Tokyo, Japan), and binocular indirect ophthalmoscopy (Keeler binocular indirect ophthalmoscope, Broomer, PA, USA; Volk pan retinal 2.2D, Mentor, OH, USA). Examination showed the left eye (OS) to be non-visual, though it did have positive direct and consensual (from left to right eye) pupillary reflexes. Additional anterior segment findings included: moderate episcleral congestion, mild diffuse corneal edema, and mydriasis. Posterior segment examination revealed asteroid hyalosis, decreased myelination and cupping of the optic nerve head, and mild retinal vascular attenuation OS. Examination of the right eye (OD) was within normal limits. IOP measured with a rebound tonometer (Tonovet, Icare USA, Raleigh, NC, USA) was 24 mmHg OD and 49 mmHg OS. Clinical findings were consistent with glaucoma OS, which, based on a lack of recognizable other ocular disease, was presumed to be primary.

**Fig. 1:**
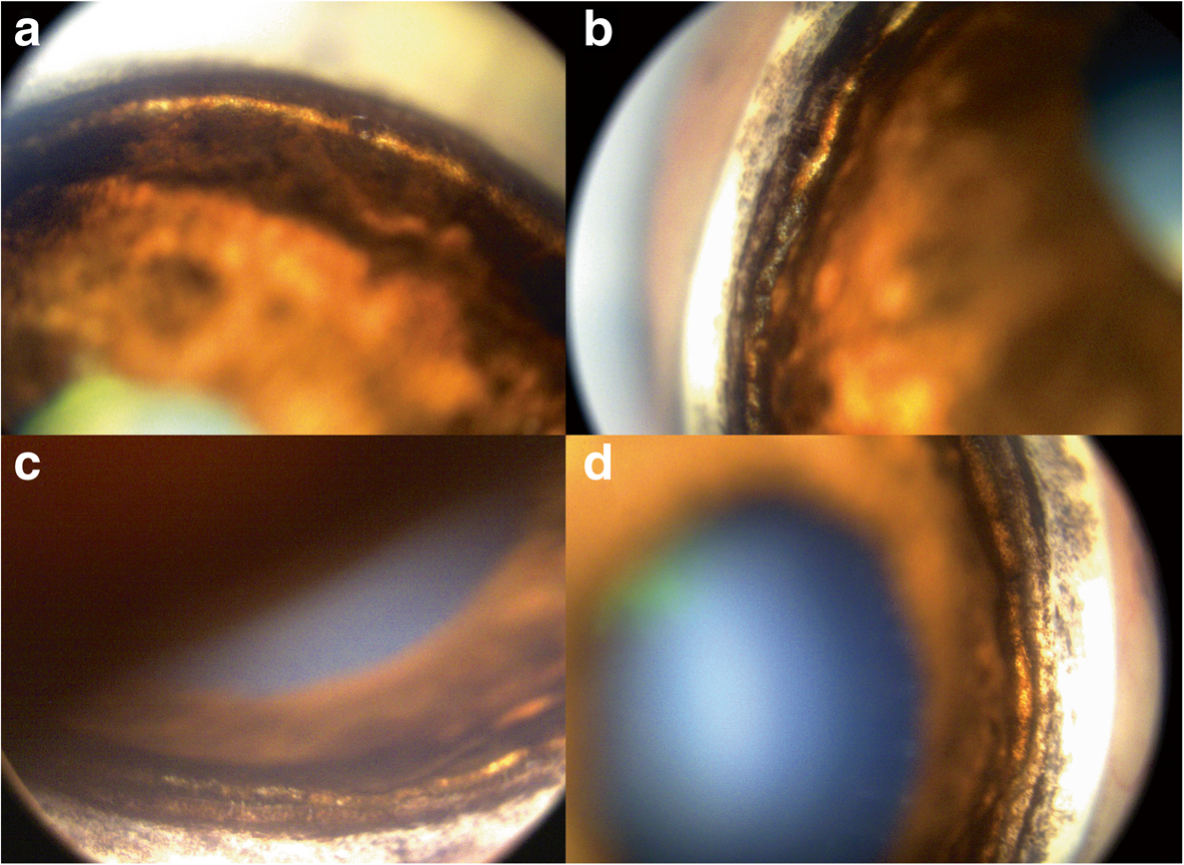
Pectinate ligament dysplasia (PLD) in a 12-year-old, neutered male Beagle with primary angle closure glaucoma (PACG). Images of superior (E), nasal (F), inferior (G), and temporal (H) ICA OD showing goniodysgenesis with moderate PLD, solid sheets, and narrow ICA

Gonioscopy was performed OD and recorded with a high-resolution ocular imaging system (RetCam, Clarity Medical Systems, Pleasanton, CA, USA). The ICA OD was narrow and had moderate pectinate ligament dysplasia (PLD) characterized by broad based pectinate ligament strands (fibrae latae) and solid sheets (laminae) throughout all 4 quadrants (Fig. 1). The ICA OS was not able to be examined due to a corneal edema. Based on the fast progressing disease process and the clinical findings, including the abnormal ICA in OD, the most likely diagnosis for OS was PACG. A blood sample was submitted for commercially available DNA testing (Optigen, Ithaca, New York, USA). The results showed that the dog did not carry the Gly661Arg missense mutation in *ADAMTS10* responsible for the only reported POAG in Beagles, further supporting the PACG diagnosis.

During the first visit, one drop of latanoprost 0.005% ophthalmic solution (Akorn, Lake Forest, IL, USA) was administered OS. Thirty minutes later, IOP OS decreased from 49 mmHg to 21 mmHg. To maintain control of the IOP OS, the patient was treated with topical glaucoma medications including latanoprost 0.005% ophthalmic solution (one drop administered OS every 12 h) and dorzolamide HCl-timolol maleate 2–0.5% ophthalmic solution (Hi-Tech Pharmacal, Amityville, NY, USA—one drop administered OS every 8 h). Based on the gonioscopy results and with hopes of delaying glaucoma onset, the OD was also prophylactically treated with dorzolamide HCl-timolol maleate ophthalmic solution (administered one drop to the left eye every 12 h) [17].

On recheck examination, one week following the initial presentation, IOPs were normal at 13 mmHg OD and 17 mmHg OS and trace aqueous flare was observed in both eyes. Menace response was positive OD, but remained negative OS. The owner elected to continue with the medical management. Thus, we recommended the same glaucoma medications at the same doses and frequencies and periodic IOP rechecks by the referring veterinarian (rDVM). The owner was also educated on how to monitor for the signs of an IOP spike including vision loss, blepharospasm, episcleral congestion, and corneal edema. IOP was well-maintained with medical management until approximately three months after initial presentation when the rDVM measured IOP OS as 32 mmHg and OD 8 mmHg. At that time, the frequency of latanoprost 0.005% ophthalmic solution was increased to every 8 h for the OS.

**Fig. 2:**
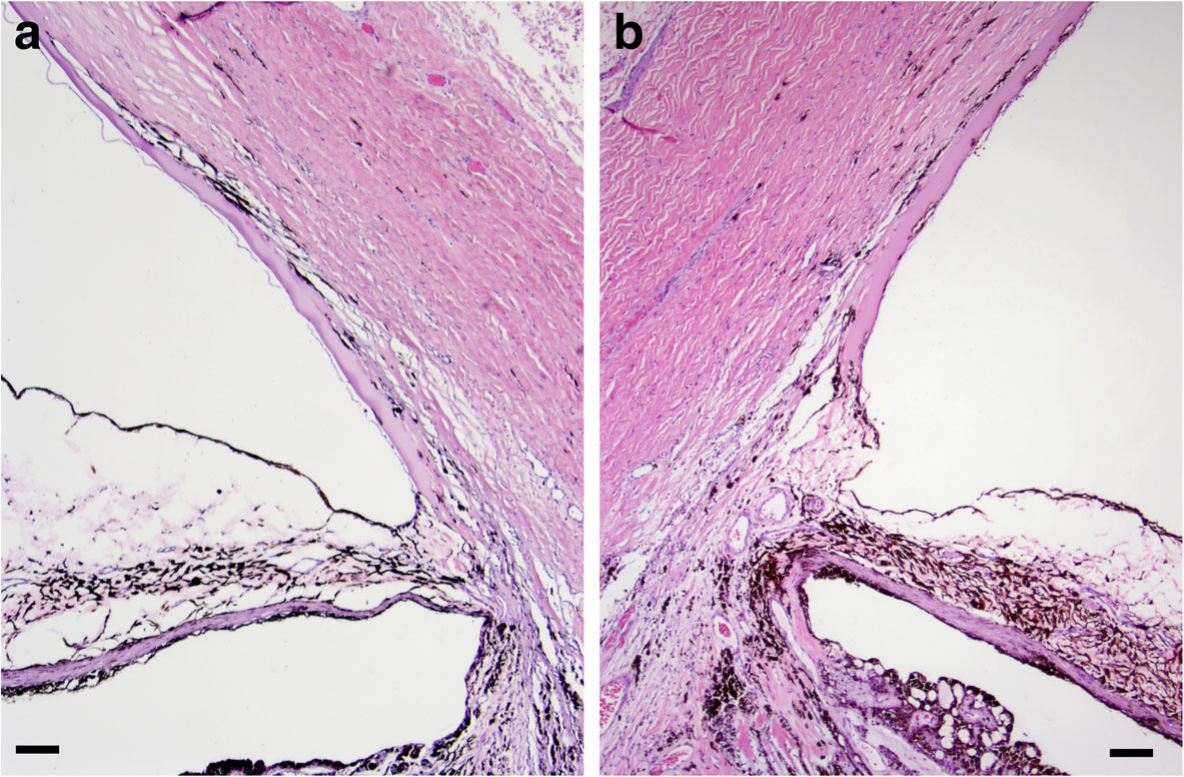
Histopathologic findings of the ICA in both eyes (OU) were consistent with goniodysgenesis. **a** OS: A broad, nonperforate, sheet-like band of uveal stroma bridges from the base of the iris to the terminal arborization of Descemet’s membrane were noted. **b** OD: Findings were comparable to OS (**a**) with the addition of a pre-iridal fibrovascular membrane (PIFM) leading to posterior synechia, mild lymphoplasmacytic anterior uveitis, and mild corneal neovascularization. Hematoxylin and eosin (H&E) stain. Scale bar in each image = 50 μm

**Fig. 3:**
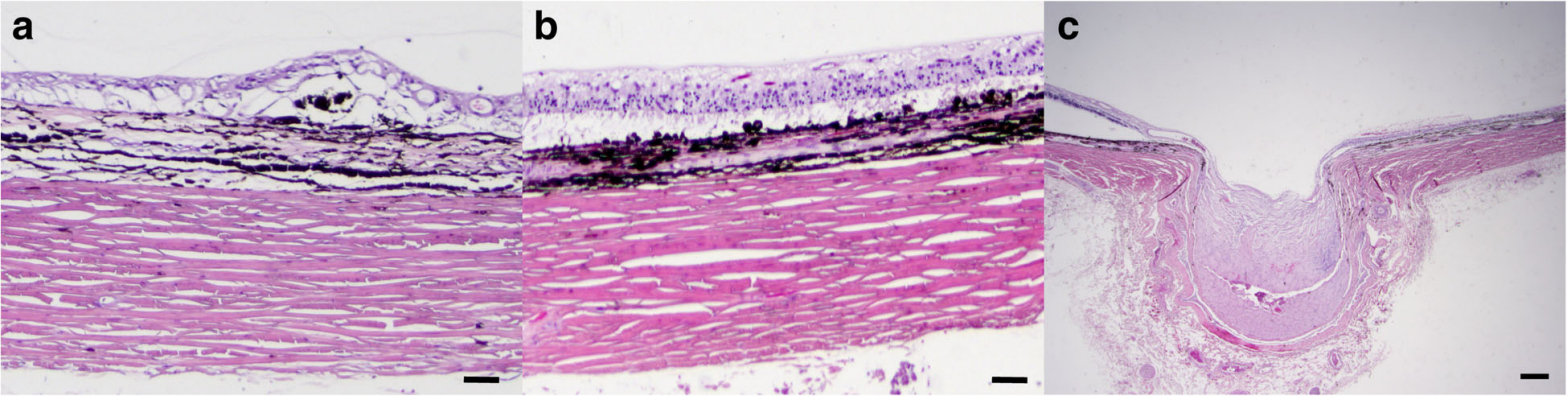
Histopathologic findings of the posterior segment OU were consistent with chronic glaucoma. **a** OS: There is severe diffuse inner to segmental full thickness retinal atrophy. In regions of full thickness retinal atrophy, which affects the nontapetal retina, there is loss of neurons from all layers, marked architectural collapse and thinning, and multifocal infiltrates of pigmented cells into the neural retina. **b** OD: There is diffuse inner to segmental full thickness retinal atrophy. In areas of inner retinal atrophy, which affects the tapetal retina, there is variable loss of neurons from the ganglion and inner nuclear layers, variable blending of the inner and outer nuclear layers, and variable rarefaction and collapse of the nerve fiber and plexiform layers. **c** OS: The optic nerve head is markedly cupped with rarefaction and mild gliosis. Cavitations within the optic nerve head contain accumulations of flocculent basophilic material, consistent with Schnabel’s cavernous atrophy. Hematoxylin and eosin (H&E) stain. Scale bar in A and B = 25 μm and C = 160 μm

Approximately six months following the initial presentation, there was another IOP spike OS to 52 mmHg; IOP OD was 20 mmHg. With OS no longer responding to topical medication, the rDVM enucleated OS for long-term pain control. Histopathologic findings OS were consistent with chronic glaucoma with goniodysgenesis. There was a broad, non-perforate, sheet-like band of uveal stroma bridging from the base of the iris to the terminal arborization of Descemet’s membrane, which was consistent with the gonioscopic findings OD (Fig. 2). The ciliary cleft OS was collapsed, the trabecular meshwork was largely unapparent, and the corneoscleral trabecular meshwork had undergone mild remodeling by loosely arranged fibrosis. In addition, there was mild pigment dispersion within the posterior chamber, inner retinal atrophy with retinal ganglion cell loss of the tapetal retina, segmental full thickness retinal atrophy of the nontapetal retina, segmental retinal detachment, marked optic disc cupping with rarefaction and mild gliosis and atrophy of the optic nerve head as well as posterior displacement of the lamina cribrosa, and mild corneal edema (Fig. 3). Based on the ophthalmic examinations, gonioscopy, genetic testing, and histopathologic evaluation, the diagnoses of PLD OD and PACG with goniodysgenesis OS were confirmed.

During the next ophthalmic examinations at MSU-VMC—performed seven months following initial presentation—IOP was 13 mmHg OD. A trace amount of aqueous flare and mild pigment deposition on the anterior lens capsule were observed OD, suggesting persistent low-grade uveitis OD. Dorzolamide HCl-timolol maleate ophthalmic solution (one drop administered to right eye every 12 h) was continued OD and a topical non-steroidal anti-inflammatory medication, diclofenac 0.1% ophthalmic solution (Akorn, Lake Forest, IL, USA, one drop to right eye every 12 h) was prescribed. Approximately 14 months from the initial diagnosis of glaucoma OS, OD progressed to acute congestive stage of glaucoma with blindness diagnosed by the MSU-VMC Emergency and Critical Care Service. Medical management failed within one week, and the owner elected to have the eye enucleated. Histopathologic findings OD were consistent with goniodysgenesis and were similar to the findings noted in OS with the addition of pre-iridal fibrovascular membrane (PIFM) leading to posterior synechia, mild lymphoplasmacytic anterior uveitis, and mild corneal neovascularization (Figs. 2 and 3). There was no retinal detachment or optic nerve cupping in this eye.

## Discussion and conclusion

This case report describes PACG with goniodysgenesis in a Beagle. PACG associated with goniodysgenesis is the most common form of primary glaucoma in dogs with reported predisposition in many breeds including the American Cocker Spaniel, Basset Hound, Chow Chow, and Boston Terrier [1, 3]. While in some veterinary ophthalmology textbooks, Beagles are listed as a predisposed breed for both POAG and PACG [17], to the best of the authors’ knowledge, this is the first peer-reviewed publication on PACG with supporting clinical and histologic data in this breed.

The patient was 12-years old at the time of diagnosis of PACG with which can be misinterpreted as progressed stage of POAG in older Beagles. However, the most distinguishable manifestation of this case from POAG reported in Beagles is the rapid onset of the clinical signs and IOP increase with a time lag of several months between the two eyes. The clinical signs of *ADAMTS10*-POAG in Beagles tends to develop more slowly over several months with progressing vision loss, mydriasis and buphthalmia, and both eyes are similarly affected [2].

Gonioscopy is an important in vivo examination method to assess iridocorneal angle and diagnose PLD [7, 18]. While PLD itself may not play a significant role in the pathogenesis of PACG, it serves as a marker for changes in the cliary cleft and trabecular meshwork [19]. However, it should be noted that while PLD is a potentially predisposing factor for glaucoma, it is not diagnostic or confirmatory for future glaucoma development. Despite this limitation, gonioscopy still has great diagnostic value in canine glaucoma. When only one eye is affected with glaucoma, gonioscopy of the other eye can, in most cases, helps differentiate primary from secondary glaucoma given that the most common form of primary glaucoma in dogs is closed angle glaucoma [1]. Even though there are no published cases of Beagles with PACG, this case suggests that the disease can occur in the breed and that gonioscopy is an important part of the clinical examination to evaluate the ICA in any glaucomatous Beagle.

POAG and PACG have different disease courses and responses to treatment. Canine POAG, such as reported in Beagles, Norwegian Elkhounds, and a number of other breeds shows very slow, and similar progression in both eyes over months and years, while it responds relatively well to medical management until the ciliary cleft collapses at the late stage of disease [8, 20–23]. On the other hand, PACG reported in many breeds including American Cocker Spaniels and Basset Hounds progresses rapidly over days and weeks, and can lead to blindness within hours to months despite aggressive treatment [1, 24]. In most PACG-affected dogs, one eye is affected first with the fellow eye developing the disease days, weeks, or months later [16]. In order to determine the prognosis and because prophylactic medical therapy has the potential to delay the onset of PACG in the second eye significantly [16], the detailed diagnostic evaluation, including gonioscopy, as performed in this case is critical.

An early-stage goniodysgenesis has been described in a 9-month-old laboratory Beagle [25]. According to this previous report [25], both eyes appeared greenish, thus increased IOP was suspected. However, with a lack of IOP data or description of visual performance, it is difficult to interpret this case report. This report also described a shallow anterior chamber with a slight conical protrusion of the iris, suggestive of anterior displacement of the iris and lens as a possible cause of narrow ICA which may have resulted in increased IOP. Possible causes of forward displacement of the iris and the lens include, retinal detachment, increased vitreal volume, and lens (sub)luxation. Anteriorly curved and displaced iris in the presented histopathology image supports this speculation. Lens (sub)luxation and retinal detachment are possible causes of secondary glaucoma in dogs [1]. Therefore, a secondary etiology cannot be completely excluded.

In fact, primary lens (sub)luxation with zonular ligament dysplasia is a frequently observed clinical sign in Beagles with POAG, often apparent before the onset of clinical POAG [14, 26]. Recently, a close/overlapping relationship between canine POAG with *ADAMTS10* mutation and canine primary lens luxation with *ADAMTS17* mutation has been suggested as the two genes play a major role in the formation of microfibrils [6, 27, 28]. In the current case, there were no signs of lens zonular damage, such as the presence of degenerated vitreous in the anterior chamber, and lens instability.

Goniodysgenesis is histologically characterized by the presence of a solid iris-like sheet of uveal stroma extending from the iris base to the termination of Descemet’s membrane [29]. With increased IOP in the acute congestive stage, the ciliary cleft collapses within 1–5 days and the sheet of uveal stroma is located anterior to the collapsed ciliary cleft [29], which were consistent with our histopathology findings in the present case. The histopathology image presented in the aforementioned report [25] demonstrates a fairly open ICA and ciliary cleft with an intact pectinate ligament without the presence of sheet-like uveal stroma. This makes it hard, for comparative ocular pathologists, to accept the diagnosis of glaucoma with goniodysgenesis.

Mild anterior uveitis has been clinically observed in both eyes of the patient. Histopathology of OS revealed chronic signs of mild lymphoplasmacytic anterior uveitis as well as PIFM formation. The possible etiologies for uveitis include but are not limited to infections, immune-mediated, metabolic, and neoplasia which all can lead to development of secondary glaucoma [30]. However, concurrent signs of uveitis also have been regularly reported in dogs and humans with PACG and this has been attributed to IOP-associated anterior segment ischemia [24, 31, 32]. PIFM formation in PACG has been previously documented [33]. It is likely the presence of vascular endothelial growth factor in the aqueous humor and VEGF receptor 1 and 2 in the iris contributes to PIFM formation in canine PACG [34, 35]. It is also possible that use of moderate frequency of latanoprost, a prostaglandin analogue, contributed to uveitis development [36, 37]. Based on these observations, it was suggested that not all glaucomas in eyes with uveitis are secondary [24]. In the present case, mild non-progressive uveitis with lack of infectious or neoplastic origins on clinical and histopathologic evaluation was most consistent with IOP-related uveitis.

Although full clinical data was not accessible, goniodysgenesis was diagnosed in another enucleated eye from a 14-year-old spayed female Beagle via histopathologic examinations at the MSU Veterinary Diagnostic Laboratory. This dog had a four-month history of glaucoma in the enucleated eye. IOP and other clinical findings for either eye were not available. The histopathologic findings were almost identical to the present case (data not shown). Considering both dogs were over 12 years, it is speculated that PACG may tend to develop in older Beagles compared to the earlier POAG onset with clinical signs becoming noticeable by the owner at 4–6 years of age [1]. More number of patients with PACG in Beagle breed will be required to confirm the late onset of the disease.

The Gly661Arg missense mutation in *ADAMTS10* is known to be responsible for POAG in Beagles [6, 15], but was ruled out as a cause for PACG in Shiba Inus, Shih Tzus, Chihuahuas, Australian Cattle Dogs, Jack Russell Terriers, Jindos, Siberian Huskies, and Yorkshire Terriers [38]. Five susceptibility genes or loci were identified as possible contributors to canine PACG: these comprise *COL1A2,* *RAB22A*, and *NEB* in Basset Hounds [39, 40], 9.5-Mb locus on canine chromosome 8 in Dandie Dinmont terriers [41], and *SRBD2* gene in Shiba Inus and Shih Tzus [42]. However, a specific disease gene has yet to be identified. DNA testing showed that the present case was not a carrier of the Gly661Arg missense mutation in *ADAMTS10* associated with POAG in Beagles. This finding further supported the PACG diagnosis in this case. It is worth to note that breeds affected by one genetic mutation resulting in glaucoma can develop a complete different mutation resulting in a similar or different phenotype of the disease.

To the authors’ knowledge, this is the first report of goniodysgenesis and PACG with supporting clinical, gonioscopic, and histopathologic data in the Beagle. It highlights the importance of gonioscopy in Beagles with glaucoma. Further studies with a larger number of dogs are warranted to characterize clinical manifestations and inheritance of PACG in this breed.

## Abbreviation

ICA: iridocorneal angle
IOP: intraocular pressure
MSU-VMC: Michigan State University, Veterinary Medical Center
OD: Oculus Dexter, right eye
OS: Oculus Sinister, left eye
OU: Ocular Utro, both eyes
PACG: primary angle closure glaucoma
PIFM: pre-iridal fibrovascular mebrane
PLD: pectinate ligament dysplasia
POAG: primary open angle glaucoma
rDVM: referring veterinarian
